# Improvement in Anthropometric Measurements of Malnourished Children by Means of Complementary Food and Nutritional Education in Fars Province, Iran: A Community-Based Intervention

**DOI:** 10.3389/fnut.2022.813449

**Published:** 2022-03-02

**Authors:** Razieh Shenavar, Seyedeh Forough Sajjadi, Azam Farmani, Mina Zarmehrparirouy, Leila Azadbakht

**Affiliations:** ^1^Department of Community Nutrition, Shiraz University of Medical Science, Shiraz, Iran; ^2^Department of Community Nutrition, School of Nutrition Sciences and Food Technology, National Nutrition and Food Technology Research Institute, Shahid Beheshti University of Medical Sciences, Tehran, Iran; ^3^Department of Community Nutrition, School of Nutritional Sciences and Dietetics, Tehran University of Medical Sciences, Tehran, Iran

**Keywords:** malnutrition, malnourished children, nutritional intervention, community intervention, children

## Abstract

**Background:**

Childhood malnutrition could have adverse impacts on the growth of child and eventually on fertility and general economic growth, and still, this issue remains a worldwide priority and a concern. This study aimed to evaluate the effectiveness of the national nutritional interventions program on the improvement and nutritional status of malnourished children children aged 6–59 months.

**Methods:**

This community-based intervention study was conducted with 1288 acute and moderately malnourished children aged 6–59 months referred to health centers. Children received combined nutritional education and counseling with the provision of affordable complementary food for 10 months. Anthropometric measurements were assessed monthly according to the standard protocols.

**Results:**

Our results showed the reduction in the risk of malnutrition among children after nutritional interventions for weight-for-height (WHZ) (*P* < 0.001), height-for-age (HAZ) (*P* < 0.001), and weight-for-age (WAZ) (*P* = 0.008). Total malnourished children indicated improvement in HAZ (<-3 SD: OR = 1.10, *P* = 0.026), WAZ (<-2SD: OR = 1.21, *P* < 0.001; <-3SD: OR = 1.60, *P* < 0.001), and WHZ (<-3SD: OR = 1.10, *P* = 0.030). Controlling potential confounders (socioeconomic status, childrens' birth supine length, and weight) showed a significant amelioration in HAZ (<-2 SD: OR = 6.20, *P* = 0.020; <-3 SD: OR = 9, *P* = 0.003) and WAZ (<-2 SD: OR = 5.85, *P* = 0.010; <-3 SD: OR = 7.50, *P* = 0.004). In urban areas, significant amelioration was observed in HAZ (<-3 SD: OR = 1.22, *P* = 0.010) and WAZ (<-2 SD: OR = 1.24, *P* = 0.003; <-3 SD: OR = 1.64, *P* < 0.001). In rural, considerable amelioration was observed in WAZ (<-2 SD: OR = 1.20, *P* = 0.010; <-3 SD: OR = 1.50, *P* < 0.001) and WHZ (<-3 SD: OR = 1.20, *P* = 0.020).

**Conclusion:**

Nutritional training and counseling as well as complementary food intervention among the malnourished children significantly improved the nutritional status of children. So community-based intervention is recommended to reduce the malnutrition among children.

## Introduction

Malnutrition in childhood is a significant risk factor for disability and death worldwide (1, 2). Although there has been a gradual decline in the prevalence of malnutrition (3), it is associated with more than 50% of all deaths in young children (4). Over the past three decades, improved diets and public health policies have resulted in a decline in the prevalence of child malnutrition in Iran (5). Nonetheless, according to the Unicef statistics, 15% of Iranian children suffer from moderate and severe short stature, and 11% suffer from moderate and severe underweight (6). The prevalence of stunting, underweight, and wasting among under-nutrition among children under 6 years of age in Fars province was estimated at 9.53, 9.66, and 8.19%, respectively (7).

An acute decrease in food consumption in children is often combined with illness, poor appetite, anorexia, and sometimes medical complications, such as feeding problems and low birth weight. These situations lead to losing weight, failure to gain weight, and increasing acute malnutrition risk (8). Malnutrition has long-term outcomes on children's intellectual abilities, economic productivity, and tends to increase the risk of non-communicable diseases, such as overweight, metabolic syndrome, hypertension, and diabetes in adulthood (5, 9, 10). However, malnutrition is reversible with refeeding and treatment of medical complications quickly (11). The incidence of malnutrition risk factors differs across different socio-cultural factors and various geographical settings (6, 10). That is, due to the variation of food patterns and enteric pathogenic burden that can affect gut function, cultural beliefs, and access to healthcare in different regions. Unfortunately, malnutrition and poverty create a defective cycle and chain that reinforce each other and worsen the situation of society in all dimensions (6, 10).

Sufficient nutrition of children in the early stages of life is necessary to warrant the proper growth of organs and their appropriate function, adequate mental growth, and the development of the immune system (5). Further, studies demonstrated the nutritional status of children is affected by various socioeconomic factors, such as education of mother, household wealth, nutritional status, and demographic characteristics (1, 12, 13). The economic status of households and maternal education were the most significant contributors to socioeconomic inequalities, such as mental health, the extent of using healthcare, and malnutrition among children (12). Over the past decade, there has been growing attention for scaling up nutrition interventions (14, 15). However, studies demonstrated that behavior changes, such as nutritional intervention and complimentary food could improve the nutrition status among children (16, 17). Heidkamp and Panjwani pointed out that further evidence is required to show the influence of nutrition education interventions on child wasting (18).

According to the healthcare protocol of Iran, all children under 60-month-old should receive primary healthcare in healthcare centers. In 2005, the healthcare centers of Iran launched a nutritional intervention to ensure adequate growth and development of all children. The Department of Community Nutrition mainly led this program in the Ministry of Health and Medical Education. Healthcare centers provide services through community health centers in rural and urban areas consisting of two major sections: supportive and collaborative. The supporting section has a targeted approach aiming at malnourished or growth-retarded children who lived in poor families and suffered from nutritional deficiencies and has been held with the corporation of the Imam Khomeini Relief Foundation (IKRF). These health centers were staffed with physicians, nutritionists, and healthcare workers who provide healthcare services, such as supplementary food, nutrition consultation, health education, mobilization for immunizations, and referral services. This study aimed to evaluate the effectiveness of nutritional education interventions programs combined with food complementary among malnutrition children living in low-income households in the urban and rural areas of Fars province, Iran.

## Materials and Methods

### Subjects

This community-based intervention study was conducted on children from 6 to 59 months old and their mothers/caregivers referred to health centers affiliated to the Shiraz University of Medical Sciences of Fars. A multi-stage cluster sampling technique was used, and all the health centers from 23 rural and urban areas of Fars province, Iran, were included in this study. The children were selected over 3 months from January to March 2017. A total of 2,800 children who attended health centers to receive healthcare services for maternal and child health were investigated, and 1,512 of them were excluded due to non-cooperation in completing the intervention or withdrawn from the study, and there were no follow-up measurements. Finally, 1,288 children were selected to assess the effectiveness of the intervention program. The malnourished or growth-retarded children were selected as the intervention group. Due to all the children who were eligible being allowed to enter the program, selecting the control group was impossible. Inclusion criteria had malnutrition status, poor economic condition, and parental willingness to participate their child in this study. Children with a physical handicap, intellectual disability, Phenylkenonuria, Down syndrome, hypothyroidism including cretinism and hyperthyroidism, diabetes mellitus, and those whose mothers did not consent to participate in the study were excluded. All parents signed their written informed consent before participation in quantitative surveys, qualitative interviews, and focus group discussions.

Semi-structured interviews were conducted at baseline with the parents or caregivers. During the interview, socio-demographic data were collected using a questionnaire on each household (e.g., sex, age, child's birth length/weight, access to an improved water source and a healthy environment, socio-economic status, and household demographics). Ten focus group discussions were undertaken monthly throughout the intervention period. Each one lasted between 20 and 30 min. Questions were asked regarding the content of the food basket and distribution methods, experience of the mother with the program and its objectives, and the effects of program on a child. The Ethics Committee approved this study protocol of Shiraz University of Medical Sciences (IR.SUMS.REC.1.3.94.598).

### Outcome Variables

Indicators of malnutrition status were derived from standardized Height-for-age (HAZ), Weight-for-age (WAZ), and Weight-for-height (WHZ) anthropometric measurements according to the WHO Child Growth Standards. Each of these outcome variables was ordinal response data with four categories (<-1 SD, <-2 SD, <-3 SD, and Failure to thrive). The WHO global database on child growth and malnutrition suggests a cut-off z score of ≤-2 to classify low HAZ (stunting), low WHZ (wasting), and low WAZ (underweight) as moderate malnutrition, and a z score of ≤-3 SD to define severe malnutrition (19). Moreover, in this study, children with a z score of ≤-1 SD, and children who were in the normal range but their growth curves were flat in more than two consecutive measurements were defined as a mil malnourished and failure to thrive, respectively. Children with outlier values of HAZ, WHZ, and WAZ <-5 SD were removed from the analysis. Trained health workers screened the curve of all registered children who attended the centers and selected the children whose growth curves were at least 1 SD below the median WHO growth standard in the last measurement.

### Anthropometric Measurements

In health centers, trained health workers measured the weight and height of children using a pediatric-scale (Seca, Germany) with 0.1 kg accuracy for under 2-year-old children and a digital weighing scale (Seca, Germany) with 0.1 kg accuracy for older ones wearing light clothes without shoes. For height measurement, children older than 2-year-old height was measured in the standing position by a vertical stadiometer (Seca, Germany), while the height of children under 2-year-old was taken in the supine position using a (horizontal) pediatric stadiometer (Seca, Germany). Both devices had equipped with an inextensible measuring tape with a sensitivity of 0.1 cm. Using the measurements, the healthcare workers plot the WAZ of children curves. Besides, expert nutritionists and the research team trained all health workers to measure and investigate the anthropometric indices.

### Intervention

The intervention program covered 10 months of the year, between April 2017 and January 2018. This study combined nutritional education with the provision of affordable complementary food to malnourished children for 10 months. The healthcare based on nutritional intervention implemented in the present study was composed of two main components: (1) delivering health and nutrition education modules monthly; and (2) providing households with locally-prepared complementary food. Complementary foods were packed into poultry (2 Kg), red meat (0.5 Kg), egg (1 Kg), white rice (1.5 Kg), yogurt (0.5 Kg), macaroni (0.7 Kg), oil (0.9 Kg), lentils (1 Kg), and pinto beans (1 Kg). Selected foods were distributed monthly to participating households by the cooperation of health centers and predetermined department store.

The complementary food distribution was initiated when the gathering baseline data, such as height, weight, and age of malnourished children finished. In addition, each month before complementary food packages were given, mothers/caregivers were invited to participate in the group-based nutritional education sessions. Every mother/caregiver was also offered a counseling (face-to-face) session monthly that provided them with the opportunity to learn about their children's requirements, breastfeeding, eating behavior/disorders in children, and dietary guidelines for children. The emphasis of group-based nutritional education on child feeding consists of the importance of time, appropriate complementary foods, such as nutrient-rich foods and meal frequency, responsive feeding, hand washing, food consistency, and preparation. Trained community healthcare workers and nutritionists provided the group-based nutritional education and individual counseling sessions. It was recognized that the proposed complementary food mix lacked fruit, vegetables, and various micronutrients. Therefore, mothers/caregivers were advised to intake the appropriate amount of fruits and vegetables during monthly distribution. All children were reassessed with follow-up visits each month after the first distribution of complementary food to measure height, weight for assessing the growth trend, and nutritional situation during the last month. Furthermore, in-service workshops were held for health workers and nutritionists by the research team to update and improve their health and nutritional knowledge.

### Socioeconomic Status Assessment

One of the inclusion criteria to select children was their socioeconomic condition of household. Malnourished/growth-retarded children are identified and screened actively through growth monitoring services of the Primary HealthCare system and introduced to the IKRF for the economic status confirmation of families from January to March 2017. This organization re-assessed the socioeconomic status of households, such as education and occupation of parents, asset ownership, housing status, residency area, and household income. The poverty line was determined <100 dollars a month by IKRF. After the nomination process, children were verified to attend the program.

### Statistical Analysis

All statistical analyses were performed using the IBM SPSS version 22.0 (SPSS, Chicago, IL, USA), and the WHO Anthro software (version 3.2.2) was used to calculate anthropometric indicators using weight, height/length, and age values. The normal distribution of data was assessed with the Kolmogorov–Smirnov test and histogram curves. The statistical tests, such as chi-squared, Fisher's exact test, Mann–Whitney U-test, and Kruskal–Wallis test, were used to assess differences between groups. The sign test was used for evaluating the mean difference change in WHZ, HAZ, and WAZ before and after the intervention. The generalized estimating equation (GEE) model with the cumulative logit link function and multinomial distribution was used to compare the differential changes of the outcomes over time (20–22). The socioeconomic variables (sex, education and occupation of parents, homeownership status, and child order), birth supine length of children, and birth weight of children were controlled in the models. Results were presented as odds ratios (ORs) and 95% CIs before and after the nutritional intervention. The level of statistical significance was p < 0.05.

## Results

### Study Population Characteristics

A total of 1,288 malnourished children (710 girls and 578 boys) were enrolled in this community-based intervention research. The nonparametric tests were performed considering that the normality assumptions of data were rejected. As shown in Table 1, participants were divided into two groups based on the region (rural and urban). The mean birth weight and birth supine length of the study participants were 2.97 Kg, (SD = 0.45) and 49.30 cm (SD = 3.1) (Table 1). The background characteristics of children, such as the education and occupation of parents, number of baskets that children received, and other socioeconomic features, are shown in Table 1.

**Table 1:** Background characteristics of children 6-59 months in urban and rural areas in Fars province (*N* = 1,288).

**Characteristics**		**Region**		**Total** **(*N =* 1288)**	***P*-value^a^**
		**Urban** **(*N =* 574)**	**Rural** **(*N =* 714)**		
Sex	Female	318 (55.4)	392 (54.9)	710 (55.1)	0.86
	Male	256 (44.6)	322 (45.1)	578 (44.9)	
Sex of household head	Female	22 (3.8)	32 (4.5)	54 (4.2)	0.17
	Male	128 (22.3)	134 (18.8)	262 (20.3)	
	Both parents	424 (73.9)	545 (76.3)	969 (75.2)	
	others	-	3 (0.4)	3 (0.2)	
Mother's education	Primary	414 (72.1)	566 (79.3)	980 (76.1)	0.01^*^
	Diploma	138 (24)	121 (16.9)	259 (20.1)	
	Academic	22 (3.8)	27 (3.8)	49 (3.8)	
Father's education	Primary	435 (75.8)	568 (79.6)	1003 (77.9)	0.08
	Diploma	131 (22.8)	130 (18.2)	261 (20.3)	
	Academic	8 (1.4)	16 (2.2)	24 (1.9)	
Mother's occupation	House keeper	532 (92.7)	643 (90.1)	1175 (91.2)	0.11
	Employee	42 (7.3)	71 (9.9)	113 (8.8)	
Father's occupation	Unemployed	111 (19.3)	200 (28)	311 (24.1)	0.000^***^
	Rancher/farmer / Part time worker	423 (73.7)	439 (61.5)	862 (66.9)	
	Fulltime worker/ Employee/ Self-employed	8 (1.4)	20 (2.8)	28 (2.2)	
	Retired	1 (0.2)	2 (0.3)	3 (0.2)	
	others	31 (5.4)	53 (7.4)	84 (6.5)	
Household size	≤2	3 (0.5)	5 (0.7)	8 (0.6)	0.17
	2–5	377 (65.7)	502 (70.3)	879 (68.2)	
	≥ 5	194 (33.8)	207 (29)	401 (31.1)	
Child order	1st	209 (36.4)	242 (33.9)	451 (35)	0.08
	2nd	205 (35.7)	300 (42)	505 (39.2)	
	3rd	117 (20.4)	117 (16.4)	234 (18.2)	
	4th +_	43 (7.5)	55 (7.7)	98 (7.6)	
Home ownership status	Householder	392 (68.3)	545 (76.3)	937 (72.7)	0.01^*^
	Rental property	152 (26.5)	135 (18.9)	287 (22.3)	
	Public (governmental)	4 (0.7)	3 (0.4)	7 (0.5)	
	Others	26 (4.5)	31 (4.3)	57 (4.4)	
Food basket	8	486 (84.7)	552 (77.3)	1038 (80.6)	0.004^**^
	9	43 (7.5)	75 (10.5)	118 (9.2)	
	10	45 (7.8)	87 (12.2)	132 (10.2)	
Birth weight (Kg)		2.96 ± 0.44	2.98 ± 0.46	2.97 ± 0.45	0.36
		3 [1,5]	3 [1,4]	3 [1,5]	
Birth length (cm)		49.2 ± 2.70	49.4 ± 3.40	49.30 ± 3.10	0.19
		49 [36,79]	50 [34,90]	50 [34,90]	

*Data are presented as n (%), Mean±SD or median [min, max]*;

a *P-values are resulted from chi-squared test, Fisher's exact test or Mann–Whitney U test*;

* *P-value <0.05*,

** *P-value <0.01*,

*** *P-value < 0.001*.

### Baseline Characteristics of Children According to Their HAZ, WAZ, and WHZ Classification

Baseline characteristics of 6–59 months children throughout categories of height-for-age (HAZ), WAZ, and weight-for-height (WHZ) are indicated in Table 2. The chi-square test showed significant relationships between sex, age, and categories of malnutrition indices. The underweight (19%) and severely underweight (11.10%) rates were higher in male children than female children (p < 0.001). Additionally, the wasted (17.80%) and severely wasted (10.60%) children with male gender were more frequent than female gender (p < 0.01), whereas the failure to thrive rate in female children was more than male children. The stunted and severely stunted children aged 24–35 months and 36–47 months were more frequent than others. The same results were observed for WAZ (p < 0.001). The Kruskal–Wallis test demonstrated the significant relationship between birth weight and length with malnutrition indices. The mean birth weight had substantial differences with HAZ (p = 0.03), WAZ (P < 0.001), and WHZ (P < 0.001). Moreover, the mean birth supine length of children was significantly different with the WAZ index (P < 0.001). The results of Table 2 are related to the indices before the intervention.

**Table 2:** Baseline characteristics of 6–59 months' children enrolled in intervention classified according to their height-for-age, weight-for-age and weight-for-height in Fars province.

**Characteristics**		**Height-for-age (HAZ)**				**Weight-for-age (WAZ)**				**Weight-for-height (WHZ)**			
		**<-1SD**	**<-2SD stunted**	**<-3SD severely stunted**	**Failure to thrive**	**<-1SD**	**<-2SD underweight**	**<-3SD severely underweight**	**Failure to thrive**	**<-1SD**	**<-2SD wasted**	**<-3SD Severely Wasted**	**Failure to thrive**
Sex n (%)	Female	169 (23.80)	98 (13.80)	70 (9.90)	373 (52.50)	271 (38.2)	123 (17.30)	34 (4.80)	282 (39.70)	228 (32.1)	104 (14.6)	45 (6.40)	333 (46.90)
	Male	138 (23.90)	87 (15.10)	73 (12.60)	280 (48.40)	201 (34.8)	110 (19)	64 (11.10)	203 (35.10)	178 (30.8)	103 (17.8)	61 (10.60)	236 (40.80)
P-value^a^		**0.31**				0.000^***^				0.008^**^			
Age (months) n (%)	6-11	4 (7.40)	-	1 (1.90)	49 (90.70)	7 (13)	5 (9.3)	-	42 (77.7)	17 (31.50)	9 (16.70)	5 (9.30)	23 (42.60)
	12-23	60 (19.20)	24 (7.70)	33 (10.60)	195 (62.50)	100 (32.1)	53 (17)	17 (5.4)	142 (45.50)	96 (30.80)	47 (15.10)	24 (7.70)	145 (46.50)
	24-35	93 (23.80)	63 (16.20)	45 (11.50)	189 (48.50)	136 (34.9)	73 (18.70)	31 (7.90)	150 (38.50)	124 (31.8)	63 (16.20)	33 (8.50)	170 (43.60)
	36-47	81 (23.90)	69 (20.40)	39 (11.50)	150 (44.20)	156 (46)	61 (18)	25 (7.40)	97 (28.60)	118 (34.8)	54 (15.90)	25 (7.40)	142 (41.90)
	48-59	69 (35.80)	29 (15)	25 (13)	70 (36.2)	73 (37.8)	41 (21.20)	25 (13)	54 (28)	51 (26.4)	34 (17.60)	19 (9.80)	89 (46.10)
P-value^a^		0.000^***^				0.000^***^				**0.94**			
Region n (%)	Urban	137 (23.90)	95 (16.60)	70 (12.20)	272 (47.40)	207 (36.1)	110 (19.20)	44 (7.70)	213 (37.10)	170 (29.6)	101 (17.6)	38 (6.60)	265 (46.20)
	Rural	170 (23.80)	90 (12.60)	73 (10.20)	381 (53.40)	265 (37.1)	123 (17.20)	54 (7.60)	272 (38.10)	236 (33.1)	106 (14.8)	68 (9.51)	304 (42.60)
P-value^a^		**0.078**				**0.84**				**0.076**			
Child order n (%)	1st	102 (22.60)	75 (16.60)	49 (10.80)	225 (49.90)	154 (34.1)	85 (18.80)	32 (7.10)	180 (39.90)	151 (33.50)	78 (17.30)	27 (6)	195 (43.20)
	2nd	128 (25.30)	73 (14.50)	59 (11.70)	245 (48.50)	186 (36.8)	96 (19)	43 (8.50)	180 (35.60)	155 (30.70)	71 (14.10)	53 (10.50)	226 (44.80)
	3rd	53 (22.60)	26 (11.10)	20 (8.50)	135 (57.70)	99 (42.3)	34 (14.50)	14 (6)	87 (37.20)	78 (33.30)	43 (18.40)	15 (6.40)	98 (41.90)
	4th +_	24 (24.50)	11 (11.20)	15 (15.30)	48 (49)	33 (33.7)	18 (18.40)	9 (9.20)	38 (38.80)	22 (22.40)	15 (15.30)	11 (11.20)	50 (51)
P-value^b^		**0.28**				**0.52**				**0.08**			
Children's birth weight mean (SD)		2.93 (0.43)	2.94 (0.39)	2.94 (0.49)	3 (0.46)	2.93 (0.43)	2.89 (0.47)	2.86 (0.48)	3.08 (0.44)	2.93 (0.45)	2.88 (0.50)	2.91 (0.45)	3.04 (0.43)
P-value^b^		0.03^*^				0.000^***^				0.000^***^			
Children's birth length mean (SD)		49.16 (3.20)	49.15 (1.97)	49.08 (4.31)	49.48 (2.97)	49.06 (2.64)	49.24 (4)	48.54 (2.57)	49.75 (3.04)	49.19 (2.74)	49.55 (4.70)	48.80 (2.55)	49.41 (2.67)
P-value^b^		**0.06**				0.000^***^				**0.37**			

*HAZ, Height-for-age; WAZ, Weight-for-age; WHZ, Weight-for-height*.

a *chi-square test*,

b *Kruskal Wallis test*;

* *P-value <0.05*,

** *P-value <0.01*,

*** *P-value < 0.001*.

*HAZ, WAZ and WHZ = > Standards used Ghana Statistical Service (GSS), Ghana Health Service (GHS) and ICF Macro (2009) Ghana Demographic and Health Survey 2008. GSS, GHS and ICF Macro, Accra*.

*SD, standard deviation*.

### Prevalence of WHZ, HAZ, and WAZ in Children Before and After Intervention

The nutritional status of the children in terms of WHZ, HAZ, and WAZ is shown in Table 3. Considering that testing the normal distribution of malnutrition indices by Kolmogorov-Smirnov test was rejected. The sign test was used to determine a consistent difference between paired observations. However, there was no significant relationship throughout the categories of WAZ and prevalence of malnourished children before and after intervention (p = 0.07), WHZ and HAZ indicated an improved situation, and this relationship was statistically significant (P < 0.001).

**Table 3:** Prevalence of WHZ, HAZ, and WAZ in children 6–59 months before and after community-based intervention in Fars province (*N* = 1288).

	**Baseline**		**End line**		***P*-value^a^**
	***n***	**%**	***n***	**%**	
**WHZ**					
<-1	406	31.5	400	31.1	0.000^***^
<-2	207	16.1	168	13	
<-3	106	8.2	70	5.4	
Failure to thrive	569	44.2	650	50.5	
**HAZ**					
<-1	307	23.8	379	29.4	0.000^***^
<-2	185	14.4	213	16.5	
<-3	143	11.1	96	7.5	
Failure to thrive	653	50.7	600	46.6	
**WAZ**					
<-1	472	36.6	493	38.3	0.07
<-2	233	18.1	212	16.5	
<-3	98	7.6	61	4.7	
Failure to thrive	485	37.7	522	40.5	

*HAZ, Height-for-age; WAZ, Weight-for-age; WHZ, Weight-for-height*.

a *chi-square test*;

*** *p-value <0.001*.

### Mean Difference Change in WHZ, HAZ, and WAZ Before and After Intervention

As shown in Table 4, there are significant differences before and after intervention for WHZ (P < 0.001), HAZ (P < 0.001), and WAZ (P = 0.008). Mean difference change in WHZ and WAZ showed an increasing trend after intervention from −1.14 to −1.00 and −1.29 to −1.25, respectively. Meanwhile, the mean difference change in HAZ revealed decreased trend from −0.88 to −1.01.

**Table 4:** Mean difference change in WHZ, HAZ, and WAZ in children 6–59 months before and after community-based intervention in Fars province (*N* = 1,288).

**Indicator**	**Baseline Mean z-score** **(SD)**	**End line Mean z-score** **(SD)**	**Mean difference change in z-score** **(SD)**	***p*-value^a^**
WHZ	−1.14 (1.38)	−1.00 (1.23)	−0.14 (1.08)	0.000^***^
HAZ	−0.88 (2.05)	−1.01 (1.62)	0.13 (1.37)	0.000^***^
WAZ	−1.29 (1.28)	−1.25 (1.07)	−0.04 (0.85)	0.008^**^
BMI	14.5 (1.83)	14.46 (1.61)	0.04 (1.44)	0.29

*HAZ, Height-for-age; WAZ, Weight-for-age; WHZ, Weight-for-height; BMI, Body mass index*.

*Mean difference = (baseline-endline)*.

a *P-values are resulted from Sign Test*.

** *P-value <0.01*,

*** *P-value < 0.001*.

### The Risk of Malnutrition Among Malnourished Children Before and After Nutritional Intervention

The GEE model results are presented in Table 5. In the total sample, the changes of stunted (<-1 SD: OR = 0.40, <-2 SD: OR = 0.73), underweight (<-1 SD: OR = 0.60), and wasted (<-1 SD: OR = 0.50, <-2 SD: OR = 0.85) over time due to the nutritional intervention were significantly lower than failure to thrive levels (p < 0.001). Whereas, the changes in severe stunted (<-3 SD: OR = 1.10, P = 0.026), underweight (<-2 SD: OR = 1.21, P < 0.001), severe underweight (<-3 SD: OR = 1.60, P < 0.001), and severely wasted (<-3 SD: OR = 1.10, P = 0.030) were considerably greater than failure to thrive levels across time. Urban children had lower changes in the mild stunted (<-1 SD: OR = 0.38), stunted (<-2 SD: OR = 0.80), underweight (<-1 SD: OR = 0.58), and wasted (<-1 SD: OR = 0.43, <-2 SD: OR = 0.80) over time than failure to thrive levels (p < 0.001). While they had more changes in severe stunted (<-3 SD: OR = 1.22, P = 0.010), underweight (<-2 SD: OR = 1.24, P < 0.01), and severe underweight (<-3 SD: OR = 1.64, P < 0.001). In addition, the children of rural areas had the same pattern in changes of malnutrition indices over time.

**Table 5:** Assessing the effects of the nutritional interventions on malnutrition indices in children 6–59 months over time using the GEE model.

**Outcome**			**HAZ**			**WAZ**			**WHZ**		
			**<-1SD**	**<-2SD**	**<-3SD**	**<-1SD**	**<-2SD**	**<-3SD**	**<-1SD**	**<-2SD**	**<-3SD**
Total sample	Crude model	OR (95% CI)	0.40 (0.33, 0.40)	0.73 (0.66, 0.80)	1.10 (0.96, 1.20)	0.60 (0.55, 0.66)	1.21 (1.10, 1.33)	1.60 (1.40, 1.70)	0.50 (0.41, 0.50)	0.85 (0.77, 0.93)	1.10 (1.01, 1.22)
		*P*-value	0.000^***^	0.000^***^	0.026^*^	0.000^***^	0.000^***^	0.000^***^	0.000^***^	0.000^***^	0.030^*^
	Adjusted model	OR (95% CI)	3.07 (0.71, 13.4)	6.20 (1.40, 27)	9 (2.02, 39.4)	2.80 (0.73, 11.20)	5.85 (1.50, 22.80)	7.50 (1.90, 29.60)	0.30 (0.10, 0.98)	0.53 (0.15, 1.80)	0.70 (0.20, 2.40)
		*P*-value	0.130	0.020^*^	0.003^**^	0.130	0.010^*^	0.004^**^	0.040^*^	0.310	0.060
Urban	Crude model	OR (95% CI)	0.38 (0.33, 0.43)	0.80 (0.70, 0.92)	1.22 (1.06, 1.40)	0.58 (0.51, 0.67)	1.24 (1.08, 1.43)	1.64 (1.41, 1.90)	0.43 (0.37, 0.49)	0.80 (0.70, 0.92)	1.04 (0.91, 1.20)
		*P*-value	0.000^***^	0.001^**^	0.010^*^	0.000^***^	0.003^**^	0.000^***^	0.000^***^	0.003^**^	0.600
	Adjusted model	OR (95% CI)	11.75 (1.20, 118.66)	25.32 (2.50, 256.70)	38.50 (3.80, 392)	1.72 (0.20, 14.80)	3.70 (0.43, 31.90)	4.92 (0.57, 42.35)	0.16 (0.03, 0.95)	0.30 (0.05, 1.83)	0.40 (0.06, 2.40)
		*P*-value	0.040^*^	0.010^*^	0.002^**^	0.620	0.230	0.150	0.040^*^	0.200	0.300
Rural	Crude model	OR (95% CI)	0.35 (0.30, 0.39)	0.67 (0.59, 0.76)	0.95 (0.83, 1.07)	0.61 (0.53, 0.69)	1.20 (1.04, 1.34)	1.50 (1.30, 1.70)	0.46 (0.42, 0.54)	0.88 (0.77, 0.99)	1.20 (1.03, 1.33)
		*P*-value	0.000^***^	0.000^***^	0.390	0.000^***^	0.010^*^	0.000^***^	0.000^***^	0.040^*^	0.020^*^
	Adjusted model	OR (95% CI)	1.51 (0.29, 7.75)	2.88 (0.56, 14.80)	4.10 (0.79, 21.10)	4.20 (0.75, 23.45)	8.22 (1.47, 45.80)	10.50 (1.90, 58.40)	0.42 (0.08, 2.15)	0.78 (0.15, 4)	1.05 (0.20, 5.40)
		*P*-value	0.620	0.210	0.090	0.100	0.020^*^	0.010^*^	0.300	0.800	0.950

*HAZ, Height-for-age; WAZ, Weight-for-age; WHZ, Weight-for-height; OR, odds ratio; 95% CI, 95% confidence interval*.

*<-1SD = mild; <-2SD = moderate; <-3SD = severe; reference category = Failure to thrive; Adjusted model = adjusted for (sex, parent's education, parent's occupation, sex of household head, home ownership status, birth length, birth weight)*.

* *P-value <0.05*,

** *P-value <0.01*,

*** *P-value <0.001*;

*significant P-values are shown in bold font*.

According to the adjusted models, children who lived in urban areas were more likely to sustain recovery throughout the follow-up period and demonstrated that malnourished children with <-3 SD severely stunted in HAZ remained statistically significant (OR = 38.50, p < 0.01). Similarly, undernourished children in WAZ and WHZ indicated the same pattern after the intervention. In the rural area, malnourished children in <-2 SD underweight (OR = 8.22) and <-3 SD severely underweight (OR = 10.50) had significant changes across time (p < 0.05). In the total sample, malnourished children in <-2 SD HAZ (OR = 6.20, p < 0.05), <-3 SD HAZ (OR = 9, p < 0.01), <-2 SD WAZ (OR = 5.85, p < 0.05), and <-3 SD WAZ (OR = 7.50, p < 0.01) had more significant changes than others.

## Discussion

In this community-based intervention research on malnourished children, we examined the effectiveness of the national nutritional interventions program on the growth and nutritional status of malnourished children under 60-month-old. However, we did not find a significant association between children's birth order and the region where lived with indicators of malnutrition status (HAZ, WAZ, and WHZ), birth weight and supine length, age, and gender of children showed a significant correlation with the prevalence of malnutrition. Moreover, the prevalence and risk of malnutrition decreased after the nutritional intervention in categories of WAZ, WHZ, and HAZ. To the best of our knowledge, this is the first community-based intervention research on malnourished children in Fars province to assess the effectiveness of complementary food combined with the nutritional education and counseling intervention on malnutrition.

We failed to find any significant association between age and the risk of malnutrition. However, age might be associated with malnutrition since age in children is a factor that can influence the nutritional status of a child (23). Children under 4 years old are highly vulnerable to malnutrition because their bodies need sufficient nutrients for development and growth; in addition, they are easily affected by diseases that can ultimately lead to malnutrition (23). In an unmatched case-control study on 182 malnourished and 189 well-nourished children in Ghana, Edem M. A. Tette et al. found that the age of 24 months or less was associated with malnutrition (8). Moreover, Kassie and Workie reported a high prevalence of malnutrition in children below the age of 4 years (24). Malnutrition could be attributable to the late introduction of supplementary food with low nutritional quality (25).

The findings of this study indicate that birth weight is associated with the prevalence of malnutrition among 6–59-month children. According to several studies, low birth weight connects with poor postnatal growth, especially during the first year of life (26, 27). Investigations identify low birth weight as a stimulus of childhood malnutrition which can contribute significantly to stunting (28). Children with a birth weight <2,500 g have more risk of being underweight compared with those with an average birth weight (>2,500 g) (29). For instance, Trivedi et al. classified low birth weight as a predictor for malnutrition, mortality, and morbidity in children (30). Similarly, Edem M. A. Tette found Children who had low birth weight, an episode of diarrhea (within the last 6 months) or showed evidence of developmental delay were associated with higher odds of malnutrition (8). Moreover, Arifeen et al. reported a study of infant growth and their associations to birth weight in low socio-economic situations in Bangladesh that indicated birth weight was the most critical determinant of subsequent growth status during infancy (31). The impact of low birth weight varies between different settings, which makes it necessary to obtain local data (27). Comparing the nutritional status among developing countries and showed that the nutritional status in developing countries is affected by several factors, such as poor economic growth, poverty, and frequency of diseases (32). However, the nutritional status in developed countries is more affected by eating disorders than poverty or economic growth (33).

In this study, the prevalence and risk of malnutrition decreased after complementary food combined with nutritional education and counseling intervention in categories of WAZ, WHZ, and HAZ. In agreement with our finding, MacFadden et al. showed that a food subsidy program could provide an essential nutritional safety net and potentially improve nutrition for young children and pregnant women living on low incomes (34). In addition, a community-based, randomized, controlled trial conducted among 605 malnourished children aged 6–9 months in Bangladesh showed that a suitable nutrition education package prevented malnutrition and growth faltering among young children (35). Menasria L. et al. revealed that there was no significant association between the provision of local foods combined with the nutritional education and counseling on the development of child nutritional status compared with nutritional education and counseling alone. However, children who received them were healthier with better energy, zinc, and iron status. These different findings may be partially due to low energy and nutrient of food supplements, a child's refusal to intake food supplements, and intestinal parasite infections in these children (16). Investigations showed improved knowledge of infant-feeding practices and preventive healthcare behaviors by the children could successfully prevent growth faltering of the young children (17, 36). In addition, education on complementary feeding alone can improve HAZ and WAZ scores and dietary intake among young children (35, 37).

Several types of research revealed evidence for designing and developing a national multidisciplinary program for improving the nutritional status of children. There are policy approaches to decrease financial barriers, healthier dietary patterns, and tackle nutritional inequalities, such as food subsidy programs. For instance, the Special Supplemental Nutrition Program for Women, Infants, and Children (WIC) in the United States (38) and Healthy Start in the United Kingdom (34) are directed toward young children and women. The national program of improving the nutritional status of children had some positive impacts on the livelihood and emotions of the families in economic difficulty. The results recommend that proper nutrition education and empowerment interventions combined with the supplemental food distribution could be more effective if its targeting strategy is more tuned and implemented. Furthermore, in families with economic difficulty, food packages should design for the entire household and adjust for household size along with some empowerment approaches (39).

This study has several limitations. First of all, nutrition counseling and education activities may have differed in terms of quality among various health centers. Therefore, it could have affected child dietary intake differently. As the food package was consumed at home, there was no evidence that all children intake the suggested amount of foods daily. This situation could have restricted the power to find out significant differences. In addition, however, in the current study, mothers/caregivers were trained for breastfeeding and support skills in individual counseling sessions, the breastfeeding status could have led to the differences observed. On the other hand, the strengths of the current study are sufficient sample size and adjustment for several potential confounders. Our study was the first to examine the effectiveness of both complementary food packages and nutrition education on malnourished children in Fars province.

## Conclusion

Based on the findings of this study, it is concluded that nutritional training and counseling, as well as food complementary among malnourished children, significantly improve the nutritional status of children improving through the HAZ, WAZ, and WHZ even after controlling for a range of potential confounding factors. This effective means of reducing malnutrition in children should be scaled-up in communities as preventive and management strategies and should be prepared in a culturally appropriate manner.

## Data Availability Statement

The original contributions presented in the study are included in the article/supplementary material, further inquiries can be directed to the corresponding author.

## Ethics Statement

The studies involving human participants were reviewed and approved by Shiraz University of Medical Sciences. Written informed consent to participate in this study was provided by the participants' legal guardian/next of kin.

## Author Contributions

LA and RS contributed to the conception and design of the study. AF organized the database. MZ performed the statistical analysis. SS wrote the first draft and sections of the manuscript. All authors contributed to manuscript revision, read, and approved the submitted version.

## Conflict of Interest

The authors declare that the research was conducted in the absence of any commercial or financial relationships that could be construed as a potential conflict of interest.

## Publisher's Note

All claims expressed in this article are solely those of the authors and do not necessarily represent those of their affiliated organizations, or those of the publisher, the editors and the reviewers. Any product that may be evaluated in this article, or claim that may be made by its manufacturer, is not guaranteed or endorsed by the publisher.
